# Impact of Drying and Storage Conditions on the Bioactive and Nutritional Properties of Malolactic Wine Lees

**DOI:** 10.3390/foods14223852

**Published:** 2025-11-11

**Authors:** Roberta Barreto de Andrade Bulos, Carolina Oliveira de Souza, Cedenir Pereira de Quadros, Otávio Augusto Durando Leme, Luiz Claudio Corrêa, Maria Beatriz Prior Pinto Oliveira, Susana Machado, Aline Camarão Telles Biasoto, Pedro Paulo Lordelo Guimarães Tavares, Renata Quartieri Nascimento, Marcelo Andrés Umsza-Guez

**Affiliations:** 1 Food Science Postgraduate Program, Faculty of Pharmacy, Federal University of Bahia, Salvador 40170-100, BA, Brazil; betab_a@hotmail.com (R.B.d.A.B.); carolods@ufba.br (C.O.d.S.); aline.biasoto@embrapa.br (A.C.T.B.); pp.lordelo@gmail.com (P.P.L.G.T.); rqnutri@gmail.com (R.Q.N.); 2 School of Pharmacy, Federal University of the São Francisco Valley (UNIVASF), Petrolina 56300-000, PE, Brazil; quadros@univasf.edu.br (C.P.d.Q.); otavio_durando@hotmail.com (O.A.D.L.); 3 Brazilian Agricultural Research Corporation, Embrapa Semi-Arid, Petrolina 56302-970, PE, Brazil; claudio.correa@embrapa.br; 4 Network of Chemistry and Technology/Associated Laboratory for Green Chemistry (REQUIMTE/LAQV), Department of Chemical Sciences, Faculty of Pharmacy, University of Porto, 4050-313 Porto, Portugal; beatoliv@ff.up.pt (M.B.P.P.O.); smachado@ff.up.pt (S.M.); 5 Brazilian Agricultural Research Corporation, Embrapa Environment, Jaguariúna 13918-110, SP, Brazil

**Keywords:** wine lees, by-product reuse, malolactic fermentation, oven, freeze-drying, amino acids

## Abstract

Wine lees, a winemaking by-product, have high potential for reuse due to their significant phenolic content and antioxidant capacity. To preserve their composition and enhance their feasibility for incorporation into food products, this study evaluated the effects of oven drying (40 °C) and freeze-drying on the physicochemical and nutritional characteristics of malolactic lees, as well as the impact of the storage time. Samples were analyzed at 0, 45, and 90 days of storage at 25 °C under light exposure conditions. Total phenolic content was determined by Folin–Ciocalteu, antioxidant activity by DPPH and FRAP assays, and phenolic groups by HPLC-DAD-FD. Both methods preserved a high protein content (~20%), with 44.66% essential amino acids and an essential amino acid index of 1.55, indicating high-quality proteins. The freeze-dried lees showed a higher phenolic content (77.92 mg GAE/g), whereas the oven-dried lees exhibited greater antioxidant activity, likely due to the formation or release of bound phenolic compounds induced by thermal processing. Among the phenolic groups, tannins were the most favored in terms of preservation. Storage time significantly influenced the parameters evaluated, indicating the impact of drying on stability. While freeze-drying better preserved phenolic compounds, oven drying was considered the most suitable option for large-scale application.

## 1. Introduction

Wine lees (WL) are an important by-product generated after the fermentation stages that take place during winemaking and are considered to be rich in bioactive compounds such as anthocyanins, flavanols, flavonols, phenolic acids, and stilbenes [1]. The presence of these compounds in WL is due to adsorption by the cell walls of the bacteria and yeasts used to ferment the must in wine production [2,3].

Phenolic compounds from WL have high antioxidant capacity and are commonly applied in the food, cosmetic, and pharmaceutical industries [4]. However, their concentration and properties can be altered during winemaking, for example, by anthocyanin hydrolysis mediated by lactic acid bacteria (especially of the genera *Lactobacillus*, *Pediococcus*, and *Oenococcus*) [5].

Despite their valuable characteristics, WL are still mainly used for low-value purposes such as animal feed and fertilizers. Valorizing WL through drying aligns with circular economy strategies by converting winery waste into functional food ingredients with nutritional and technological potential [6,7].

To enable their application as functional ingredients, different drying techniques have been tested for WL [8]. Among the various conventional drying methods, oven drying is the most common due to its simplicity, low cost, ease of implementation, and recommendations for large-scale production. It is a process based on convective heat transfer: heated air promotes the evaporation of moisture from the material. However, this technique often causes thermal degradation of bioactive compounds [9]. As an alternative, freeze-drying, a technique that operates at low temperatures and acts through the sublimation of the water present in the material, has been widely studied as it offers superior protection to thermosensitive compounds, thus preserving nutritional properties—despite its higher energy cost and processing time [10,11]. Dried WL has been used by several researchers to enrich food such as cookies, cereal bars, and ice cream. In this type of food formulation, WL have the ability to improve nutritional value, act as a natural colorant, and add antioxidant properties, due to the presence of phenolic compounds [12,13].

In the food context, another important point is storage time, which represents a critical factor for the stability of the bioactive compounds present in by-products such as WL, as environmental factors such as light, oxygen, and humidity can significantly compromise the shelf life of the processed material [14]. Although several studies indicate the benefits of applying powders from wine by-products to food ingredient in terms of preservation and extending shelf-life, few studies have addressed the stability of WL powder itself during storage [15,16]. One reason for this gap is related to the variability in the composition of WL, which depends on factors such as grape variety, winemaking process, and malolactic fermentation conditions, making it difficult to establish standardized storage protocols [2]. Additionally, the complexity of WL’s composition poses another challenge for this type of research, since the high concentration of phenolic compounds, proteins, polysaccharides, and yeast cells makes it highly susceptible to oxidation reactions, enzymatic browning, and microbiological changes during storage [1]. Therefore, generating reliable shelf-life data that support the development of functional ingredients and nutraceuticals derived from WL for the food and pharmaceutical industries is essential for advancing circular economy strategies in the wine sector.

Considering that the drying process can affect the physicochemical profile of foods, this study aims to investigate the influence of oven drying at a temperature of 40 °C and freeze-drying on red WL, regarding the presence of phenolic compounds, antioxidant activity, physicochemical properties, the nutritional profile, and, additionally, the effect of the storage time of the WL powder over a period of 90 days.

## 2. Materials and Methods

### 2.1. Materials and Reagents

The WL used for the study, a by-product of the winemaking process during the malolactic fermentation stage in stainless steel tanks with grapes of the Cabernet Sauvignon variety, were provided by a winery located in the São Francisco Valley/Pe region, Brazil (9°23′34″ S, 40°30′28″ O). The WL were stored in a freezer at −20 °C until dried.

The reagents used for total phenolic compounds and antioxidant activity were, respectively, absolute ethyl alcohol (Labsynth—Diadema, SP Brazil) and Folin–Ciocalteu solution, gallic acid, 2,2-diphenyl-1-picrylhydrazyl [DPPH], and 2,4,6-Tris [2-pyridyl]-1,3,5-triazine [TPTZ] (Sigma-Aldrich—St. Louis, MO, USA). The individual phenolic compound standards described in Section 2.4.7 (Sigma-Aldrich—St. Louis, MO, USA) used for the high-performance liquid chromatography (HPLC), and the solvents ethanol, methanol, acetonitrile, and phosphoric acid (Merck—São Paulo, SP, Brazil), were of chromatographic grade.

### 2.2. Preparation of the Wine Lees Powder

For the preparation of the wine lees powder, the operating parameters were determined based on preliminary data from the literature [9,15]. The WL were filtered through a conventional sieve to remove the skins, seeds, and stalks, followed by centrifugation (5200 rpm, 4 °C) for 15 min to separate the supernatant. The precipitate was divided into two parts: one dried in an oven with forced air circulation (model TE-394/1, Tecnal, Piracicaba, SP, Brazil) at 40 °C for 48 h and the other freeze-dried (L101 Freeze Dryer, Liobras, São Carlos, SP, Brazil) for 72 h. Both samples were ground in a domestic grain grinder (model 80,393 Hamilton Beach, Glen Allen, VA, USA) until they reached homogeneous granulometry to form powders. All the analyses using the WL powders were carried out in triplicate.

### 2.3. Obtaining the Alcoholic Extract

The oven-dried and freeze-dried wine lees were extracted in a 1:5 (*w*/*v*) ratio using ethanol (80% *v*/*v*) and stirred for 2 h at room temperature [17]. The solids were removed by filtering through a paper filter, and the liquid filtrate was subsequently concentrated in a rotary evaporator at 55 °C for 1 h. The extracts obtained were analyzed for antioxidant activity, as well as the total content, identification, and quantification of phenolic compounds.

### 2.4. Analysis Methods

#### 2.4.1. Color Characteristics CIE L*a*b*

A colorimeter (CR 5 model, Konica Minolta, Tokyo, Japan) in reflectance mode with a 30 mm diameter aperture was used to obtain the L*, a*, and b* values using the CIE (International Commission on Illumination) scale. The L* parameter corresponds to luminosity or brightness, the a* coordinate ranges from green (−60) to red (+60), and b* ranges from blue to yellow, from −60 to + 60, respectively. Δ*E* corresponds to the magnitude of the color difference between the samples and was calculated from Equation (1):

(1) $$\Delta E\;=\;\sqrt{\left\{(\Delta L*{)}^{2}+(\Delta a*{)}^{2}+(\Delta b*{)}^{2}\right\}}$$

#### 2.4.2. Proximate Composition

The physicochemical parameters were determined using the official methods described by AOAC (2005) [18]. All analyses were carried out in triplicate. Moisture content was determined by oven drying (model TE-394/I, Tecnal, Piracicaba, SP, Brazil) at 105 °C ± 5 °C until a constant weight was obtained. The total ash content was determined after incinerating the samples in a muffle furnace (model 402-D, Lavoisier, São Paulo, SP, Brazil) at 550 °C. The lipid content was determined by Soxhlet extraction using petroleum ether as the solvent. The total nitrogen content was determined using the Kjeldahl method, and the crude protein content was calculated using a factor of 6.25. The ANKOM system was used to determine the total crude fiber content, and the total carbohydrate or NIFEXT fraction (nitrogen-free fraction) was calculated as the difference between the total dry mass (100%) and the sum of the percentages of the moisture, lipid, ash, protein, and crude fiber contents.

#### 2.4.3. Amino Acid Profile and Protein Quality

To determine the amino acid profile of the samples, the methodology described by [19] was used. Acid hydrolysis (HCl 6M—110 °C/24 h) was carried out to quantify the amino acids, and alkaline hydrolysis (KOH 4M) to determine tryptophan. The internal standard used was Norvaline (2 mg/mL). The profiles of free and total amino acids were analyzed on an integrated high-efficiency chromatography system using LC-NetII/ADC hardware, a binary pump (PU-980, Jasco, Tokyo, Japan), an autosampler (AS-4150 RHPLC autosampler, Jasco, Tokyo, Japan), a multiwavelength detector (MD-2015 Plus, Jasco, Tokyo, Japan), a fluorescence detector (FP-2020 Plus, Jasco, Tokyo, Japan), and an oven (Model 7981, Hengoed, UK). An automatic pre-column derivatization procedure combining two derivatization reagents (OPA/3-MPA and FMOC) was used in the sampler. The separation of the amino acids was carried out on a ZORBAX Eclipse Plus C18 column (4.6 × 250 mm, 5 μm), maintained at 40 °C. The mobile phase gradient system consisted of (A) phosphate/borate buffer (10 mM Na_2_HPO_4_: 10 mM Na_2_B_2_O_7_ (pH = 8.2):5 mM NaN_3_) and (B) MeOH:ACN: H_2_O (45:45:10, *v*/*v*/*v*), as follows: 0.85 min, 2% B; 33.4 min, 57% B; 33.5 min, 85% B; 39.3 min, 85% B; 39.4 min, 2% B; 40.0 min, 2% B (flow rate: 1.5 mL/min). Fluorescence detection was monitored at λ excitation = 340 nm/λ emission = 450 nm (from 0.0 to 26.2 min) for OPA derivatives and at λ excitation = 266 nm/λ emission = 305 nm (from 26.2 to 40.0 min) for FMOC derivatives. At the same time, OPA derivatives were monitored at 338 nm and FMOC derivatives at 262 nm.

Amino acids were grouped into essential and non-essential. Protein quality was assessed according to the essential amino acid index (EAAI) and amino acid score (AAS) [20]. The EAAI was calculated according to Equation (2) and the AAS according to Equation (3).

(2) $$EAAI=\sqrt[{9}]{\frac{\left(mg\;of\;amino\;acid\;in\;1g\;of\;tested\;protein\right)}{\left(mg\;of\;amino\;acid\;in\;1g\;of\;reference\;protein\;*\right)}x\left(etc\;**\;for\;all\;essential\;amino\;acids\right)}$$

(3) $$AAS\;(\%)=\frac{\left(mg\;of\;amino\;acid\;in\;1g\;of\;tested\;protein\right)}{\left(mg\;of\;amino\;acid\;in\;1g\;of\;reference\;protein\;*\right)}\times 100$$

* Value obtained from the amino acid score recommended by the Dietary Protein Quality Evaluation in Human Nutrition. Report of an FAQ Expert Consultation FAO/WHO Expert Consultation on Protein and Amino Acid Requirements to calculate the quality of a protein to be consumed by children over 3 years old, adolescents, and adults.

** Repeat the calculation until the formula is complete with all the essential amino acids.

#### 2.4.4. Stability of Wine Lees Powder for 90 Days

The experimental design evaluated the stability of the material as a function of storage time and light, based on the content of total phenolic compounds, antioxidant activity, quantification, and identification of individual phenolic compounds by HPLC-DAD-FD and color.

Wine lees powder was stored in an oven at approximately 25 °C and analyzed at predetermined time intervals of 0, 45, and 90 days. The 90-day storage period was selected based on typical shelf-life evaluation protocols for food ingredients and previous studies on phenolic compound stability in dried fruit by-products. The samples were stored in plastic containers and divided into two experimental groups: with light exposure (white Lorenzetti LED lamp, 9 watts, 806 lumens) and without light, the latter being protected from light incidence by wrapping with aluminum foil.

#### 2.4.5. Total Phenolic Compounds

The content of total phenolic compounds was determined by spectrophotometry (model Lambda 900 UV/VIS, Perkin Elmer^®^, Norwark, CT, USA), according to the Folin–Ciocalteu method [17,21], using an absorbance of 765 nm. The analytical calibration curve was constructed using gallic acid as a standard. The results are expressed in mg of gallic acid equivalent/g (mg GAE/g).

#### 2.4.6. Antioxidant Activity

The antioxidant activity of the samples was determined using the alcoholic extract described above. Two methods were used: (1) Evaluation of antioxidant activity by the free radical DPPH [22], and the results obtained were expressed as EC_50_ (concentration of antioxidant required to inhibit 50% of the free radical) in μg/mL. (2) Reduction of iron (III) to iron (II) by the action of antioxidants (FRAP), with results presented in μM ferrous sulfate equivalents per g of dried extract [8]. For both methods, the results were measured in a spectrophotometer at an absorbance of 517 nm and 595 nm, respectively (model Lambda 900 UV/VIS, Perkin Elmer^®^, Norwark, CT, USA).

#### 2.4.7. Individual Phenolic Compounds by HPLC-DAD-FD

The compounds were separated, identified, and quantified by HPLC as described by [23]. A Waters chromatograph (model 269, Alliance System, Waters Corporation, Milford, MA, USA) was used, equipped with a reverse phase column (150 × 4.60 mm 3 μm, Gemini-NX C18, Phenomenex, Torrance, CA, USA), pre-column (4.0 × 3.0 mm, C18, Phenomenex, Torrance, CA, USA), diode array detectors (DAD), and fluorescence detectors (FLD). The ethanolic extracts were filtered through a 0.45 µm pore membrane and injected in triplicate with an injection volume of 10 µL. The mobile phases consisted of a 0.85% (*v*/*v*) solution of ortho-phosphoric acid in water (phase A) and acetonitrile (phase B) at a flow rate and oven temperature of 0.5 mL/min and 40 °C, respectively. The gradient used started at 100% A (0 min) and adjusted to 93% A and 7% B (10 min); 90% A and 10% B (20 min); 88% A and 12% B (30 min); 77% A and 23% B (40 min); 65% A and 35% B (45 min); and 100% B (55 min), followed by a 5 min re-equilibration at initial conditions (100% A) before the next injection.

The compounds were identified by comparison of retention times and UV-Vis spectra with authentic standards and confirmed by co-injection (spiking) with the corresponding standards. Quantification was performed using external calibration curves prepared with authentic standards at concentration ranges of 0.5–100 μg/mL (r^2^ > 0.995 for all compounds). Limits of detection (LOD) and quantification (LOQ) ranged from 0.01 to 0.50 μg/mL and 0.05–1.50 μg/mL, respectively. The DAD detector was used to monitor the following: at 280 nm, gallic acid, epicatechin (-)-gallate, epigallocatechin (-)-gallate, and cis-resveratrol; at 320 nm, trans-resveratrol, caffeic acid, syringic acid, ferulic acid, chlorogenic acid, caftaric acid, piceatannol, and viniferine; at 360 nm, kaempferol-3-O-glucoside, myricetin, rutin, isoquercetin, and isorhamnetin-3-O-glucoside; at 520 nm, all anthocyanins (cyanidin-3-glucoside, cyanidin-3,5-diglucoside, malvidin-3,5-di-O-glucoside, malvidin-3-glucoside, delphinidin-3-glucoside, peonidin-3-O-glucoside). FLD was used with excitation at 280 nm and emission at 320 nm for (+)-catechin, procyanidin B1, procyanidin B2, procyanidin A2, and (-)-epicatechin. The results were expressed as mg of compound per g of dried extract (mg/g) [24].

#### 2.4.8. Statistical Analysis

The data were subjected to analysis of variance, and the means were compared using Student’s *t*-test, as well as ANOVA and Tukey’s test, at a significance level of 5%. The software used was STATISTICA 7.0. Principal component analysis (PCA) was determined using PAST software, version 4.03 (University of Oslo, Norway), to assess the correlation between color parameters and anthocyanins.

## 3. Results and Discussion

### 3.1. Characterization of the Wine Lees

Evaluating the yield between the drying methods, the dried wine lees showed 41.8% for oven drying at 40 °C and 43.8% for freeze-drying, respectively. These comparable yields indicate that both processes were effective in moisture removal and solid concentration, resulting in similar recovery of dry matter for subsequent compositional and bioactive analyses.

The results of the proximate composition are shown in Table 1. The moisture content differed significantly between treatments: 5.00 ± 0.20 g/100 g for freeze-dried wine lees and 8.50 ± 0.20 g/100 g for oven-dried samples (*p* < 0.00), which is consistent with the findings of other authors for grape pomace [25,26]. Both results are below the maximum moisture limit of 15% established by RDC N^o^.711 (ANVISA). These results indicate that freeze-drying enhances water removal efficiency, thereby improving product stability and shelf life.

Moisture corresponds to the water content of the food and directly influences its stability. A lower moisture content helps the preservation of the food’s antioxidant activity by reducing microbial growth, oxidation, and hydrolysis of antioxidant compounds, as well as decreasing the water activity in the sample, since there will be fewer water molecules available for chemical reactions in the system, allowing for long periods of storage [25].

The lower moisture content of the freeze-dried sample likely contributed to higher dry-basis nutrient concentrations compared to oven drying [25]. Regarding the ash content in the dried wine lees, the freeze-dried samples showed a higher value than the oven-dried ones (14.70 ± 0.02 g/100 g and 12.86 ± 0.03 g/100 g, *p* < 0.01). However, the opposite occurred for crude fiber (4.90 ± 0.20 g/100 g for oven-dried WL and 3.60 ± 0.40 g/100 g for freeze-dried WL, *p* < 0.03), and this difference can be explained by the formation of compounds analogous to fibers, such as melanoidins, during the use of higher temperatures in oven drying [20,27].

There is controversy about the fiber content of wine lees in general. Some studies report a significant fraction of this parameter, while others claim that it is not significant. This divergence is due to the different analytical methods applied (Van Soest gravimetric; enzymatic-gravimetric; enzymatic-chemical; among others), winemaking technologies, and factors associated with the cultivation and variety of grapes used in the production of wines [28].

The different drying methods did not affect the composition of the samples in terms of protein and carbohydrate content, since the values found were not statistically different (*p* > 0.05). Similar results have been reported in previous studies using grape waste destined for the production of wine juice [8,28].

For both dried wine lees in this study, the amount of carbohydrates was significant, greater than 50%, which can be explained by the fact that fructose and glucose were not fully metabolized by the microorganisms during fermentation and remained in the grapes, being transferred to their by-products [25]. Similarly, the protein concentrations identified in the samples (20.30 and 19.93 g/100 g) proved to be quantitatively relevant, higher than the values reported in previous studies using different wine by-products: 9.85 to 11.92 g/100 g for pomace and 12.12 g/100 g for grape seeds [29,30]. This result is associated with the autolysis process of the yeasts during fermentation, as well as the lactic acid bacteria involved in malolactic fermentation [28].

The lipid content present in the WL was statistically different between the oven-dried and freeze-dried samples (1.20 ± 0.07 and 2.02 ± 0.08 g/100 g, *p* < 0.00, respectively). This difference can be attributed to the distinct mechanisms of each drying process. During oven drying at 40 °C, prolonged exposure to elevated temperatures may promote lipid oxidation and the volatilization of certain lipid fractions, resulting in lower lipid retention. In contrast, freeze-drying operates under vacuum and at low temperatures, which minimizes lipid degradation [15]. Additionally, this difference is possibly due to both grape skin contribution and microbial lipids, as lipids are essential components of microbial cell membranes.

### 3.2. Quantification of Total Phenolics

The concentrations of total phenolics in the WL powders differed statistically between the drying methods used (Table 2). At time zero, the freeze-dried WL had a phenolic compound content 23.42% higher than the concentration of the sample dried in an oven at 40 °C. During freeze-drying, the cellular structure of the wine lees can be broken down by the ice crystals formed, promoting better extraction of the phenolic content [31]. In contrast, under the oven drying, the application of heat promotes thermal degradation of thermolabile phenolic compounds, particularly anthocyanins and flavonols, through deglycosylation and oxidation reactions [11,32]. Also, prolonged exposure to oxygen during convective drying facilitates non-enzymatic oxidation of phenolics, further reducing their extractability.

Previous studies comparing both drying methods through the analysis of different matrices have also reported that conventional drying techniques that use temperature to remove the water present in the sample show lower results than those achieved by freeze-drying [32].

The importance of other factors for the results of this parameter, such as the stage of collection of the lees studied, is reinforced in the literature by other authors who evaluated the total phenolic content in Pinot Noir and Riesling wine lees using different winemaking techniques [33]. Among the values achieved, some are similar to those reported in this work (72.6 and 68.7 mg GAE/g).

Table 2 also shows the results for the total phenolic content during the 90-day storage period. It was found that the samples showed different behaviors in terms of the preservation of phenolic compounds, varying according to exposure to light and storage time. Linear regression was also carried out to obtain an R^2^, where the values ranged from 0.8891 to 0.9834, indicating a strong linear correlation.

The possible photodegradation and oxidation of the samples was observed, processes in which light and storage outside a refrigerated environment accelerate the decomposition of phenolic compounds, causing changes in their molecular structure and compromising their stability [34,35].

Other studies on wine by-products and foods have confirmed that exposure to light and storage time significantly affect the stability of total phenolics. Research reveals variations in the levels of these compounds during storage, highlighting the importance of controlling the environment to preserve the bioactive properties of these materials [17,34].

At 45 days, oven-dried samples exposed to light (58.20 ± 0.00 mg GAE/g, *p* < 0.04) showed slightly higher total phenolic content than samples without light (57.30 mg GAE/g). This can be explained by light catalyzing the release of bound phenolics from thermally weakened cell walls and the formation of intermediate oxidation products detected by the Folin–Ciocalteu method, temporarily increasing apparent phenolic content [35]. The subsequent decline at 90 days (52.10 ± 0.01 vs. 55.80 ± 0.08 mg GAE/g, *p* < 0.05) confirms that photodegradation ultimately predominates [35].

### 3.3. Antioxidant Activity

The antioxidant activity was evaluated using DPPH and FRAP methods (Table 3 and Table 4). At time zero, oven-dried WL powder (40 °C) exhibited lower EC_50_ values (37.02 ± 0.00 μg/mL, *p* < 0.03) compared to freeze-dried samples (42.12 μg/mL ± 0.00 μg/mL, *p* < 0.03), indicating superior antioxidant efficacy despite lower total phenolic content. This suggests that thermal processing may release bound phenolic compounds from the cell wall matrix or promote the formation of compounds with enhanced antioxidant properties [8,36]. Partial oxidation and structural modification of phenolic compounds may enhance their radical scavenging activity, even when the total phenolic content decreases [37].

These mechanisms suggest that the higher antioxidant activity observed in oven-dried samples results not only from native phenolics but also from thermally generated or transformed compounds, highlighting the complex interplay between composition and functionality induced by heat treatment. In particular, heat exposure may promote the depolymerization of high-molecular-weight tannins into smaller, more active phenolics, and trigger Maillard-type reactions between amino acids and reducing sugars, forming melanoidins and other nitrogen-containing compounds with strong reducing and radical scavenging capacity [38,39]. Similar behavior has been reported in other thermally processed plant matrices, where increased antioxidant capacity was attributed to the formation of such Maillard-derived products or partially oxidized phenolics, even as total phenol levels declined [37,40].

Linear regression (R^2^) was performed between the points, and it was found that storage time explains between 96.21% and 98.55% of the variability observed in antioxidant activity by the DPPH method. It demonstrates a consistent linear relationship of decline in this functional property over the period evaluated, with similar behavior between the different processing treatments and storage conditions.

In general, the results presented for WL powder are similar to those seen in a previous study of grape skins, in which other authors found EC_50_ values ranging from 25.1 to 41.1 μg/mL [41].

FRAP results corroborated the DPPH findings at time zero, with oven-dried samples showing higher reducing capacity (1075.52 ± 0.00 μM ferrous sulfate/g) than freeze-dried samples (829.55 ± 0.00 μM ferrous sulfate/g).

Antioxidant activity is associated with the levels of phenolics present in wine lees, since they act by inhibiting free radicals or chelating metals [42]. However, studies have also linked the freeze-drying technique and its ability to promote the isomerization of some compounds that directly influence the antioxidant activity of the sample, such as carotenoids [43,44]. Freeze-drying can also increase the porosity of the sample, leaving these compounds more exposed to oxygen and, thus, degradation [36].

When evaluating the results related to storage time, a behavior similar to that previously described for the samples was observed for the DPPH method: despite the reduction in phenolic content, antioxidant activity increased. This phenomenon aligns with mechanisms including the formation and release of antioxidant compounds during thermal processing, structural modifications in phenolic compounds, greater availability for free radical reactions, and compound complexation processes [38,39]. The FRAP results showed a contrasting reaction: the freeze-dried samples exhibited progressive antioxidant decline, while oven-dried samples showed an initial decrease followed by recovery, consistent with previous findings [17]. Methodological differences explain these divergences: DPPH measures radical scavenging capacity while FRAP assesses the reduction in potential via Fe^3+^ to Fe^2+^ conversion [45]. FRAP demonstrates superior reproducibility, and DPPH’s purple coloration may cause spectral interference in pigmented matrices like WL [46].

WL applications in foods such as hamburgers and ice cream offer enhanced antioxidant capacity, protecting against lipid and protein oxidation while replacing additives like sodium ascorbate [16,47].

### 3.4. Identification of Phenolic Compounds

The HPLC-DAD-FD analysis results (Table 5) revealed that WL possessed high phenolic content from the grapes due to yeast and bacteria adsorption capacity during fermentation [1,48]. Freeze-dried samples exhibited statistically higher concentrations across all groups: phenolic acids (4.40%), flavonols (29.11%), tannins (61.60%), and anthocyanins (57.24%). This superior preservation has been consistently reported in comparative studies [32], attributed to vacuum-assisted water removal that minimizes structural damage and retains original bioactive characteristics [49].

Tannins (epicatechin gallate, epigallocatechin gallate, catechin, epicatechin, procyanidin A2, B1, B2) predominated in both samples (64.04% freeze-dried; 60.81% oven-dried), with higher extraction yields because of their lower polarity in alcoholic media [50]. Procyanidin B2 was the major compound (26.05 mg/g freeze-dried; 17.01 mg/g oven-dried). Resveratrol (cis and trans forms) occurred in trace levels (respectively: 1.16 and 0.03 mg/g for oven-dried WL and 1.50 and 0.05 mg/g for freeze-dried WL), as observed in Cabernet Sauvignon wines, since these compounds are primarily transferred to wine during manufacturing [1,51,52].

The concentration of anthocyanins (cyanidin-3-glucoside, cyanidin-3,5-diglucoside, malvidin-3,5-di-O-glucoside, malvidin-3-glucoside, delphinidin-3-glucoside, peonidin-3-O-glucoside) reached 15.60 mg/g (freeze-dried) versus 8.93 mg/g (oven-dried). High temperatures significantly reduce anthocyanin stability [32,53], while vacuum application combined with low temperatures interrupt biological reactions effectively [54]. Stainless steel fermentation vessels better preserve anthocyanin content compared to wooden barrels [5]. Malvidin-3-glucoside was the majority compound in the anthocyanin group (Supplementary Table S1), as reported in previous studies [55,56].

Flavonoids, the class comprising the flavonol subgroups (piceatannol, trans-resveratrol, cis-resveratrol, viniferin, kaempferol-3-O-glucoside, quercetin-3-B-O-glucoside, isorhamnetin, myricetin, rutin), are susceptible to degradation when subjected to thermal drying methods. Experimental evidence shows that freeze-drying has less of a degradative impact on these phenolic compounds when compared to other dehydration techniques [57]. Myricetin, quercetin, isorhamnetin, and viniferin (Supplementary Table S1) predominated in all the WL, regardless of the drying method. Myricetin and quercetin stood out in a study of wine lees from the BRS Violeta variety [58]. The authors evaluated lees dried at 50 °C and freeze-dried, obtaining values that were statistically different. In general, quercetin is recognized as a very prevalent phenolic compound in wine lees, accounting for up to 85% of flavonols in Sauvignon and Cabernet Franc wine lees [6,59].

Despite the statistical difference in the concentration of phenolic acids (gallic acid, caffeic acid, caftaric acid, ferulic acid, coumaric acid) between the samples—8.03 mg/g for the sample dried at 40 °C and 8.40 mg/g for freeze-dried—the difference in the levels detected was not as significant as for the other groups. This is because thermal processes promote greater release of phenolic acids by breaking bonds with cellular compounds in the matrix [60]. For both forms of drying, gallic acid showed the highest values, as did studies using WL produced with different grape varieties [6].

**Figure 1:**
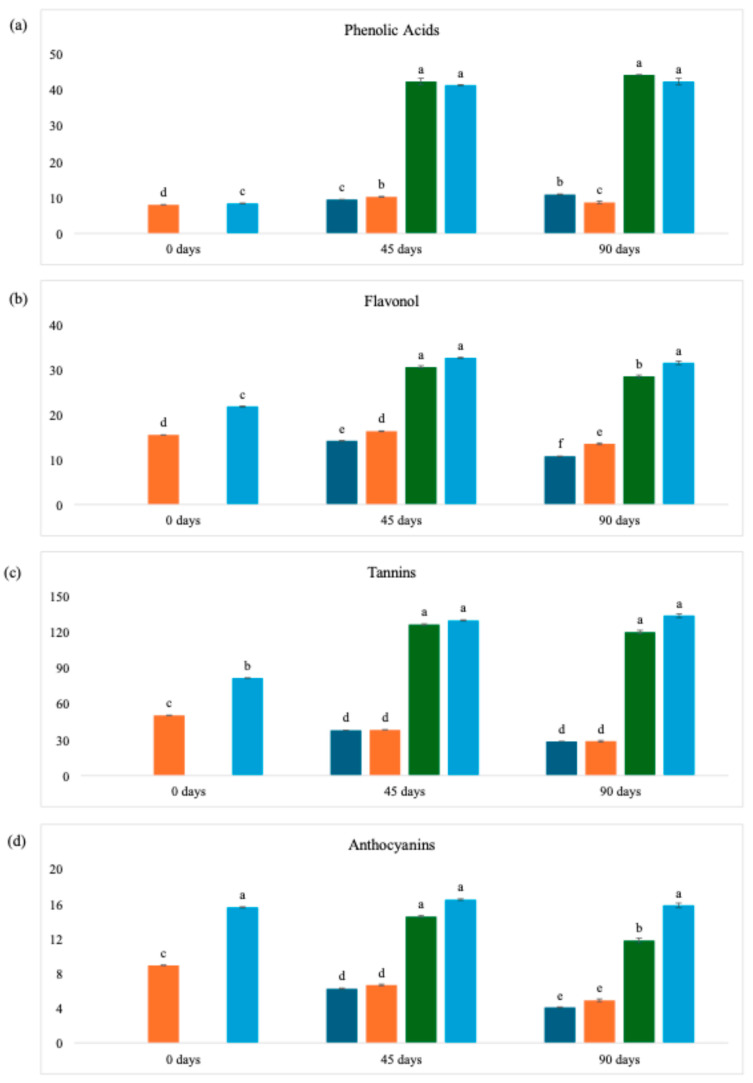
Concentration of the group of phenolic compounds identified and quantified in the wine lees powder over the storage period. (**a**) Phenolic acids; (**b**) Flavonol; (**c**) Tannins; (**d**) Anthocyanins. Dark blue bars = 40 °C (with light); Orange bars = 40 °C (without light); Green bars = Freeze-dried (with light); Light blue bars = Freeze-dried (without light). Different lowercase letters imply statistical differences (*p* ≤ 0.05) between the same samples with different types of drying according to Tukey’s test. The results were expressed in mg/g.

Figure 1 presents the phenolic groups identified by HPLC analysis. Significantly higher concentrations were observed in wine lees subjected to freeze-drying compared to oven-drying, a difference that persisted throughout the storage period (0, 45, and 90 days). This trend indicates that freeze-drying better preserves phenolic content, confirming the drying method’s decisive role in maintaining these bioactive compounds over time.

Light exposure (white LED lamp, 9 W, 806 lumens) showed no significant effect on the phenolic groups analyzed, suggesting good stability of these compounds under the tested conditions. However, the 90-day storage period may not capture long-term photodegradation. Practically, while drying strongly influences compound preservation, specific light protection may not be critical for maintaining phenolic integrity within the evaluated timeframe.

Over the course of 90 days, the stability observed for WL powders, in general, may be associated with the formation of protein–polyphenol complexes, as seen in a previous study using rice bran [61]. Corroborating this stability, another recent study found no significant changes in the total flavonoid content of honeysuckle extracts (*Lonicera japonica Thunb.*) subjected to light irradiation, showing that flavonoid compounds have good stability under these conditions, a characteristic possibly shared by the phenolic compounds present in WL powder [62].

### 3.5. Color Analysis

It can be seen in Table 6 that the color parameter L*, which corresponds to luminosity, has a lower value for wine lees dried in an oven at 40 °C. This is because the sample remains in contact with ambient air during drying, making it susceptible to oxidation [63,64]. This decrease in brightness is also associated with darkening from non-enzymatic reactions of the sample when exposed to high temperatures [63].

The a* parameter, which indicates chromaticity in the green-red direction, is correlated with the anthocyanins present in wine lees, especially malvidin-3-glucoside, and both showed the same behavior for their results: higher values with a statistical difference for freeze-dried wine lees [64].

Some studies have shown that freeze-dried foods have lower b* values than those found with temperature drying methods [11]. However, the b* result for the sample that underwent the freeze-drying technique in this study (2.37) was statistically the same as that obtained by the oven-dried WL (2.12), which appears to contradict previous findings. This discrepancy may be attributed to the specific chromatic composition of malolactic wine lees, which are characterized by a high concentration of red-purple anthocyanins and tannins, which dominate the color profile in both the L* and a* dimensions, thereby masking potential differences in the b* parameter [64]. Additionally, the relatively low absolute b* values observed for both samples (<2.5) suggest that yellow pigmentation is minimal in WL, unlike in matrices such as carotenoid-rich fruits and vegetables where heat-induced isomerization and degradation significantly impact b* values [43].

**Figure 2:**
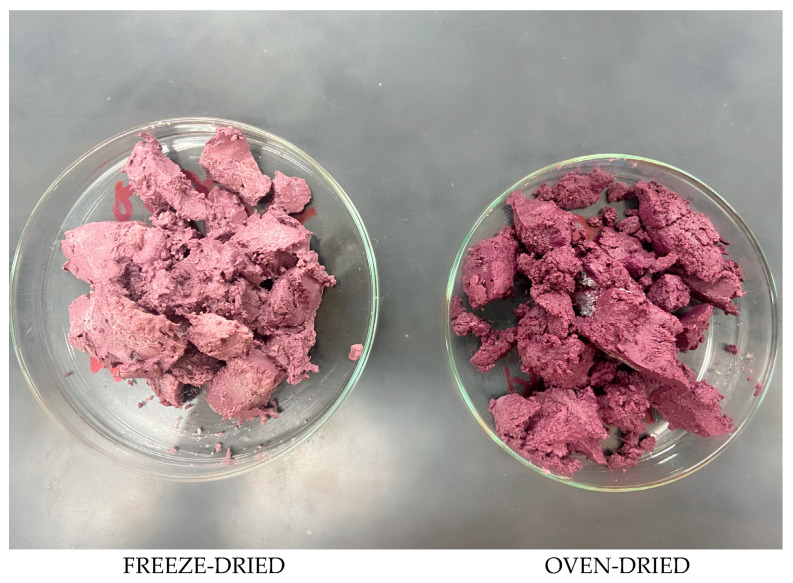
Freeze-dried and oven-dried wine lees.

The magnitude of the color difference between the WL powders dried by different methods (Δ*E*) was 6.08. This color difference can be analytically classified as follows: very different for Δ*E* > 3; different for 1.5 < Δ*E* < 3; little difference for Δ*E* < 1.5 [65]. In addition, samples with Δ*E* > 1.0 generally have differences that are perceptible to human vision, as shown in Figure 2 [66]. Therefore, it can be said that the freeze-dried and oven-dried samples at 40 °C in this study showed very different color results, capable of being noticed by an observer.

**Figure 3:**
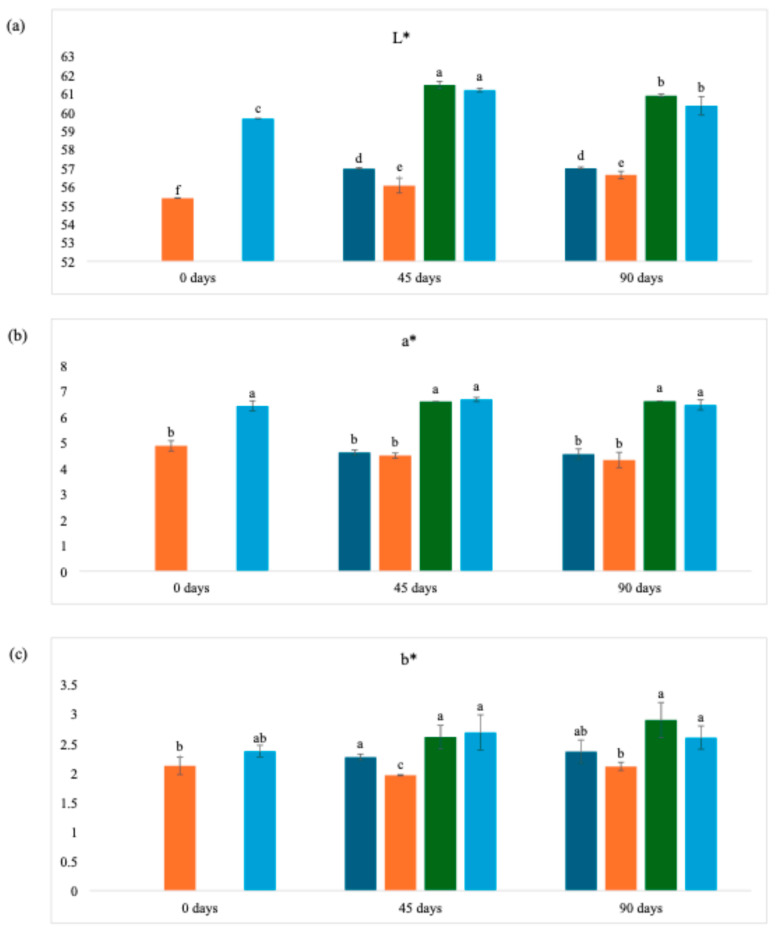
Color analysis parameters of wine lees powder over storage time. (**a**) Lightness; (**b**) Green-to-Red; (**c**) Blue-to-yellow. Dark blue bars = 40 °C (with light); Orange bars = 40 °C (without light); Green bars = Freeze-dried (with light); Light blue bars = Freeze-dried (without light). Different lowercase letters on the same line imply statistical differences (*p* ≤ 0.05) between the same samples with different types of drying according to Tukey’s test.

The color parameters throughout the storage period (Figure 3) remained the same, with the L* parameter always showing the highest values, while the b* parameter recorded the lowest values. It can also be seen that exposure to light did not significantly affect these parameters. However, the different drying methods applied to WL had a considerable influence on the results, indicating that the initial processing is more decisive for chromatic stability than the subsequent storage conditions.

### 3.6. Principal Component Analysis

**Figure 4:**
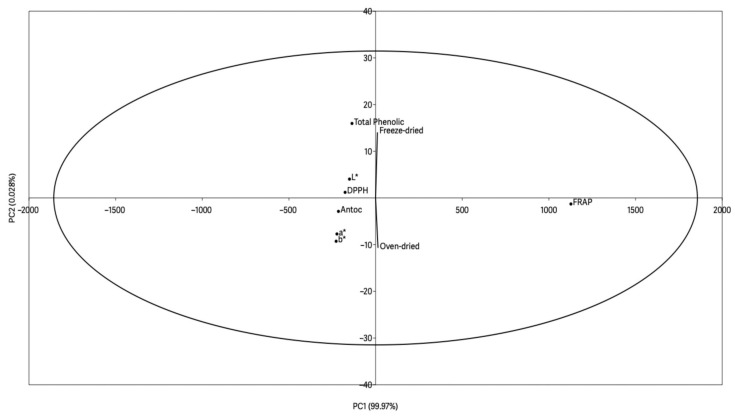
Principal component analysis of the wine lees powder.

Figure 4 shows the data from the principal component analysis between the parameters of color, anthocyanin content, phenolic compounds, and antioxidant activity at time zero. The analysis had an explanation of 99.99%, with 99.971% for PC1 and 0.028% for PC2.

The points for total phenolics and antioxidant activity (DPPH and FRAP) were grouped together in the middle-upper region of the graph, suggesting positive correlations between these parameters. This association was to be expected, since phenolic compounds are mainly responsible for the antioxidant capacity of wine by-products [67].

There was also a spatial separation between the samples processed by oven at 40 °C and freeze-drying. The freeze-dried sample was close to the bioactive compounds (total phenolics), suggesting that this processing method favors the preservation of these compounds, which is of great relevance from a technological point of view, since the preservation of antioxidant activity represents a crucial factor for the application of this by-product as a functional ingredient in the food industry [17]. On the other hand, the sample subjected to oven drying showed greater proximity to certain color parameters, indicating a possible influence of heat treatment on the powder’s chromatic characteristics.

The anthocyanin content can be directly correlated to color parameters, as they are known as natural pigments, and are generally responsible for the hue of wine and its by-products since they are transferred from the grape to the must during maceration and fermentation [68]. Anthocyanins have an inversely proportional relationship to the L* parameter, i.e., the higher the concentration of anthocyanins in the sample, the lower the luminosity [64].

### 3.7. Amino Acid Profile and Protein Quality

The amino acid profile was determined only for the freeze-dried wine lees powder, as both drying methods showed no significant difference in protein content (*p* > 0.05). Therefore, this analysis was performed to establish the reference nutritional composition of the powder at time zero, before any storage-related transformations occurred.

The amino acid profile present in the dried WL powder is shown in Table 7. Considering that both samples, freeze-dried and oven-dried at 40 °C, showed similar results for protein content, with no statistical difference between them, only the amino acid analysis of the freeze-dried WL was carried out.

Amino acids act as the main energy source for lactic acid bacteria during the malolactic fermentation of wine, establishing a direct relationship between the quality and quantity of these nitrogenous compounds and the efficiency of the fermentation rate of the must. This metabolic process not only sustains bacterial activity but also plays a crucial role in defining the sensory characteristics of the wine, since fermentation promotes significant changes in the concentration of the amino acids originally present in the must, as well as the formation and transformation of aromatic compounds [69,70].

As for the essential amino acids, leucine, lysine, valine, and phenylalanine were the ones found in greatest quantity in the dried wine lees (103.94; 72.39; 61.49, and 58.88 mg/g of protein, respectively). Leucine and valine are part of the group of branched-chain amino acids (BCAAs), commonly associated with the nutritional quality of foods, as they are involved in protein synthesis and are effective in promoting health and well-being [69]. The essential amino acids found in the lowest concentrations were tryptophan and methionine (7.14 and 13.45 mg/g of protein, respectively), a result similar to that previously described for juices made from Cabernet Sauvignon grapes [71].

Analyzing the group of non-essential amino acids, glutamic and aspartic acids were found in higher concentrations than the other amino acids (113.17 and 104.11 mg/g, respectively), a behavior similar to that seen in a previous study for wines before and after malolactic fermentation [72]. Among the amino acids identified in freeze-dried WL, arginine and proline stand out as the most abundant in wines and their derivatives, with proline being the only one not capable of being metabolized by microorganisms [69].

The ratio of essential amino acids to non-essential amino acids (EAA:NEAA) was 0.80, which is above the minimum proposed by the Energy and Protein Requirements (0.60), and between the expected range for traditional protein sources such as pork (0.67 to 0.97) [73].

The FAO/WHO [73] recommends that essential amino acids represent at least 40% of total amino acids in food; in wine lees, this value was higher (44.66%). This proportion influenced the EAAI, an index that reflects overall protein quality by comparing essential amino acids to a reference protein recommended by FAO/WHO for individuals over 3 years old. The EAAI obtained (1.55) indicates high-quality protein (values >0.90) and exceeds that of foods typically recognized as protein sources, such as silver carp fish [74,75]. Previous studies also report that wine by-products have a valuable amino acid profile, rich in essential amino acids, and can be used as nutritional enhancers in food formulation [10,13,60].

Table 8 shows the results for the Amino Acid Score (AAS), which is the rate (%) of adequacy of each essential amino acid in the protein studied in relation to the standard reference score recommended by the FAO/WHO [73]. In general, the AAS is associated with the quality of the proteins present in dry WL powder, but less accurately when compared to the EAAI [76].

All essential amino acids in dried WL powder showed AAS values above the FAO/WHO/UNU [77] reference standard of 100% [75], indicating its potential as a protein-rich supplement or ingredient in food formulations. The only exception was the group of sulfur amino acids, as cysteine was not detected, making this the limiting amino acid group.

Therefore, the amino acid profile results of this study reinforce that wine lees powder can be incorporated into traditional food formulations in order to increase its protein content and nutritional value with high-quality amino acids.

## 4. Conclusions

This study demonstrated that drying methods markedly affects the physicochemical and nutritional characteristics of malolactic wine lees. Freeze-drying better preserved the phenolic compounds and color stability, while oven drying at 40 °C yielded powders with higher antioxidant activity. From an economic standpoint, oven drying proved to be substantially more cost-effective, with operational expenses approximately 3–5 times lower than freeze-drying, primarily due to reduced energy consumption and shorter processing requirements. Considering its lower processing cost and comparable nutritional quality, oven drying emerges as a more feasible option for large-scale applications. Regardless of the method, the powders retained high protein levels and a balanced essential amino acid profile, reinforcing their potential as sustainable ingredients to enrich foods nutritionally and technologically. From a broader perspective, the valorization of this winemaking by-product contributes to sustainable food production and circular economy strategies by transforming industrial waste into functional ingredients. Future studies should investigate the incorporation of wine lees powder into various food matrices, assess sensory properties and consumer acceptance, and evaluate long-term stability under real storage conditions.

## Figures and Tables

**Table 1:** Composition of wine lees powder obtained from different drying methods.

Parameters (g/100 g)	Samples		*p* Value
	Oven	Freeze-Drying	
Moisture	8.50 ± 0.20 ^a^	5.00 ± 0.20 ^b^	0.00
Protein	20.30 ± 0.40 ^a^	19.93 ± 0.50 ^a^	0.08
Lipids	1.20 ± 0.07 ^b^	2.02 ± 0.08 ^a^	0.00
Carbohydrate	52.24 ± 0.35 ^a^	54.75 ± 0.20 ^a^	0.30
Ash **	12.86 ± 0.03 ^b^	14.70 ± 0.02 ^a^	0.01
Crude fiber	4.90 ± 0.20 ^a^	3.60 ± 0.40 ^b^	0.03

Values are expressed as arithmetic mean ± standard deviation (n = 3). Different lowercase letters on the same line imply statistical differences (*p* ≤ 0.05) between the same samples with different types of drying according to Student’s *t*-test. ** Results are expressed in g/100 g.

**Table 2:** Total phenolic content present in wine lees powder under different conditions for 90 days.

Drying Methods and Storage Conditions	Total Phenolic Content (mg GAE/g) at Different Storage Time (Days)			
	0	45	90	R^2^
40 °C with light	59.67 ^A^* ± 0.00	58.20 ^aA^* ± 0.00	52.10 ^bB^* ± 0.01	0.8891
40 °C without light		57.30 ^bA^* ± 0.01	55.80 ^aB^* ± 0.08	0.9834
Freeze-dried with light	77.92 ^A^* ± 0.08	72.20 ^bA^* ± 0.01	59.90 ^bB^* ± 0.05	0.9574
Freeze-dried without light		75.98 ^aA^* ± 0.03	63.45 ^aB^* ± 0.00	0.9574

Values are expressed as arithmetic mean ± standard deviation (n = 3). Lowercase letters in the same column imply statistical differences (*p* ≤ 0.05) between the same drying method with and without light. Capital letters in the same column imply statistical differences (*p* ≤ 0.05) between the two drying methods under the same lighting conditions according to Student’s *t*-test. Asterisks (*) indicate statistical differences (*p* ≤ 0.05) between storage times according to Tukey’s test. Results are expressed in mg GAE/g. R^2^ = coefficient of determination of the linear regression.

**Table 3:** Antioxidant activity using the DPPH method for wine lees powder dried in different conditions and stored with or without light.

Drying Methods and Storage Conditions	Antioxidant Activity (μg/mL) at Different Storage Time (Days)			
	0	45	90	R^2^
40 °C with light	37.02 ^B^* ± 0.00	35.01 ^aA^* ± 0.00	31.48 ^aB^* ± 0.01	0.9755
40 °C without light		35.20 ^aA^* ± 0.10	34.26 ^bA^* ± 0.00	0.9672
Freeze-dried with light	42.12 ^A^* ± 0.00	37.11 ^aB^* ± 0.03	33.84 ^aB^* ± 0.02	0.9855
Freeze-dried without light		40.01 ^bA^* ± 0.05	35.69 ^bA^* ± 0.01	0.9621

Values are expressed as arithmetic mean ± standard deviation (n = 3). Different lowercase letters in the same column imply statistical differences (*p* ≤ 0.05) between the same drying method with or without light. Different capital letters in the same under the same lighting condition imply statistical differences (*p* ≤ 0.05) between the two drying methods under the same lighting conditions according to Student’s *t*-test. Asterisks (*) refer to statistical differences (*p* ≤ 0.05) between storage times according to Tukey’s test. R^2^ = coefficient of determination of the linear regression.

**Table 4:** Antioxidant activity by the FRAP method for wine lees powder.

Drying Methods and Storage Conditions	Antioxidant Activity ^1^ at Different Storage Time (Days)			
	0	45	90	R^2^
40 °C with light	1075.00 ^A^* ± 0.00	341.84 ^bB^* ± 0.00	287.46 ^bB^* ± 0.00	0.8015
40 °C without light		363.03 ^aB^* ± 2.10	314.27 ^aB^* ± 0.59	0.7979
Freeze-dried with light	829.54^B^* ± 0.00	653.05 ^bA^* ± 1.21	566.51 ^aA^* ± 1.44	0.9625
Freeze-dried without light		669.37 ^aA^* ± 0.33	542.00 ^bA^* ± 1.15	0.9957

Values are expressed as arithmetic mean ± standard deviation (n = 3). Lowercase letters in the same column imply statistical differences (*p* ≤ 0.05) between the same drying method with and without light. Capital letters in the same column imply statistical differences (*p* ≤ 0.05) between the two drying methods under the same lighting conditions according to Student’s *t*-test. Asterisks refer to statistical differences (*p* ≤ 0.05) between storage times according to Tukey’s test. ^1^ The results were expressed as μM ferrous sulfate/g. R^2^ = coefficient of determination of the linear regression.

**Table 5:** Phenolic group compounds identified and quantified in the wine lees powder at time zero.

Phenolic Groups	Concentration Expressed in mg/g for Different Drying Method	
	40 °C	Freeze-Dried
Phenolic acids	8.03 ± 0.05 ^b^	8.40 ± 0.07 ^a^
Flavonol	15.51 ± 0.00 ^b^	21.88 ± 0.00 ^a^
Tannins	50.40 ± 0.00 ^b^	81.73 ± 0.00 ^a^
Anthocyanins	8.93 ± 0.03 ^b^	15.60 ± 0.02 ^a^

The values are expressed as the arithmetic mean and standard deviation (n = 3) and correspond to the sum of the compounds identified for each group. Different lowercase letters on the same line imply statistical differences (*p* ≤ 0.05) between the same samples with different types of drying according to Student’s *t*-test.

**Table 6:** Color analysis parameters of the wine lees powder at time 0.

Parameters	Sample		*p* Value
	40 °C	Freeze-Dried	
L*	55.40 ± 0.01 ^b^	59.68 ± 0.00 ^a^	0.00
a*	4.89 ± 0.01 ^b^	6.44 ± 0.00 ^a^	0.00
b*	2.12 ± 0.02 ^a^	2.37 ± 0.02 ^a^	0.06
ΔE*	6.08 ± 0.01		

Values are expressed as arithmetic mean ± standard deviation (n = 3). Different lowercase letters on the same line imply statistical differences (*p* ≤ 0.05) between the same samples with different types of drying according to Student’s *t*-test.

**Table 7:** Amino acid profile of the proteins presented in the freeze-dried wine lees powder.

Amino Acids	(mg/g Protein)
Aspartic acid	104.11 ± 2.59
Glutamic acid	113.17 ± 2.54
Arginine	56.11 ± 5.00
Alanine	63.76 ± 1.24
Glycine	53.94 ± 1.25
Hydroxyproline	12.33 ± 0.53
Proline	62.87 ± 4.66
Serine	56.02 ± 1.43
Tyrosine	31.93 ± 0.82
Phenylalanine *	58.88 ± 1.74
Histidine *	30.21 ± 0.99
Isoleucine *	49.12 ± 0.84
Leucine *	103.94 ± 2.08
Lysine *	72.39 ± 3.29
Methionine *	13.45 ± 0.38
Threonine *	49.14 ± 0.87
Tryptophan *	7.14 ± 0.32
Valine *	61.49 ± 1.51
ΣEAA	445.76 ± 7.88
ΣNEAA	554.24 ± 17.57
ΣEAA/ΣNEAA	0.80 ± 0.01
ΣAA	1000.00
EAAI	1.55 ± 0.02

* Essential amino acids; ΣEAA: Sum of essential amino acids. ΣNEAA: Sum of non-essential amino acids. ΣAA: Total sum of amino acids. EAAI: Essential Amino Acid Index.

**Table 8:** Amino acid score (AAS) of the proteins presents in the freeze-dried wine lees powder.

Amino Acids	AAS (%)
	Freeze-Dried
Histidine	188.83 ± 7.82
Threonine	199.83 ± 1.60
Valine	153.73 ± 1.50
Tryptophan	108.31 ± 6.12
Phenylalanine	143.62 ± 1.56
Isoleucine	163.75 ± 1.53
Leucine	170.39 ± 0.75
Lysine	150.81 ± 7.63
SAA *	58.50 ± 1.63
AAA **	238.96 ± 2.49

Results presented as a percentage of amino acid adequacy in relation to the recommendation for adolescents and adults. * SAA—sulfur amino acids (methionine + cysteine). ** AAA—aromatic amino acids (phenylalanine, tryptophan and tyrosine).

## Data Availability

The original contributions presented in this study are included in the article or Supplementary Materials. Further inquiries can be directed to the corresponding author.

## Supplementary Materials

The following supporting information can be downloaded at: https://www.mdpi.com/article/10.3390/foods14223852/s1, Table S1. Phenolic compounds identified and quantified in wine lees.

## Abbreviations

The following abbreviations are used in this manuscript:

AA	Amino Acids
AAA	Aromatic Amino Acids
AAS	Amino Acid Score
ANVISA	National Health Surveillance Agency
AOAC	Association of Official Analytical Collaboration
BCAA	Branched-Chain Amino Acids
CIE	International Commission on Illumination
DAD	Diode Array Detector
DPPH	2,2-Diphenyl-1-picrylhydrazyl
EAA	Essential Amino Acids
EAAI	Essential Amino Acid Index
FAO	Food and Agriculture Organization
FD	Fluorescence Detection
FRAP	Ferric Reducing Antioxidant Power Assay
GAE	Gallic Acid Equivalent
HPLC	High-Performance Liquid Chromatography
NEAA	Non-essential Amino Acids
PCA	Principal Component Analysis
RDC	Resolutions of the Collegiate Board
SAA	Sulfur Amino Acids
UNU	United Nations University
WHO	World Health Organization
WL	Wine lees
