# Supplementary material for: Impact of Drying and Storage Conditions on the Bioactive and Nutritional Properties of Malolactic Wine Lees
Source: Foods. 2025 Nov 11;14(22):3852. doi: 10.3390/foods14223852 (PMC12651660; doi:10.3390/foods14223852)
Supplement: Supplementary file 1 [file foods-14-03852-s001.zip › foods-3949807-supplementary.pdf]

Table S1.

Parameters	Samples (mg/g)	
	**40°C**	**Freeze-dried**
Gallic acid	6.81± 0.19	7.88± 0.32
Caffeic acid	1.09± 0.05	0.28± 0.39
Caffeic acid	0.00	0.00
Ferulic acid	0.13± 0.07	0.25± 0.07
Coumaric acid	0.00	0.00
**Phenolic acids**	**8.03 ± 0.00**	**8.40 ± 0.00**
Piceatanol	0.21± 0.02	0.38± 0.04
Trans-resveratrol	0.03± 0.00	0.05± 0.00
Cis-resveratrol	1.16± 0.02	1.50± 0.07
Viniferine	3.71± 0.19	5.10± 0.21
Caempferol-3-O-glucoside	0.08± 0.00	0.13± 0.11
Quercetin-3-B-O-glucoside	3.25± 0.00	4.78± 0.11
Isorhamnetin	3.10± 0.07	4.53± 0.11
Myricetin	2.73± 0.00	3.53± 0.18
Rutin	0.14± 0.02	0.10± 0.07
**Flavonol**	**15.51 ± 0.00**	**21.88 ± 0.00**
Epicatechin gallate	0.43± 0.00	0.80± 0.07
Epigallocatechin gallate	5.46± 0.05	9.83± 0.46
Catechin	9.88± 0.14	16.05± 0.57
Epicatechin	8.76± 0.41	13.88± 0.25
Procyanidin A2	0.84± 0.02	1.30± 0.14
Procyanidin B1	8.03± 0.32	13.83± 0.04
Procyanidin B2	17.01± 0.65	26.05± 0.28
**Tannins**	**50.40 ± 0.00**	**81.73 ± 0.00**
Cyanidin-3-glucoside	0.05± 0.00	0.08± 0.04
Cyanidin-3,5-diglucoside	0.05± 0.00	0.13± 0.04

Malvidin-3,5-di-O-glucoside	0.05± 0.04	0.10± 0.07
Malvidin-3-glucoside	7.28± 0.14	12.78± 0.11
Delphinidin-3-glucoside	0.38± 0.00	0.45± 0.07
Peonidin-3-O-glucoside	1.13± 0.04	2.08± 0.11
**Anthocyanins**	**8.93 ± 0.03**	**15.60 ± 0.02**
